# Trehalose ameliorates peritoneal fibrosis by promoting Snail degradation and inhibiting mesothelial-to-mesenchymal transition in mesothelial cells

**DOI:** 10.1038/s41598-020-71230-4

**Published:** 2020-08-31

**Authors:** Taito Miyake, Norihiko Sakai, Akira Tamai, Koichi Sato, Yasutaka Kamikawa, Taro Miyagawa, Hisayuki Ogura, Yuta Yamamura, Megumi Oshima, Shiori Nakagawa, Akihiro Sagara, Yasuyuki Shinozaki, Tadashi Toyama, Shinji Kitajima, Akinori Hara, Yasunori Iwata, Miho Shimizu, Kengo Furuichi, Shuichi Kaneko, Takashi Wada

**Affiliations:** 1 Division of Nephrology, Kanazawa University Hospital, Kanazawa, 920-8641 Japan; 2 Division of Blood Purification, Kanazawa University Hospital, 13-1 Takara-machi, Kanazawa, 920-8641 Japan; 3 Department of System Biology, Institute of Medical, Pharmaceutical and Health Science, Kanazawa University, 13-1 Takara-machi, Kanazawa, 920-8641 Japan; 4 Department of Nephrology and Laboratory Medicine, Institute of Medical, Pharmaceutical and Health Sciences, Kanazawa University, Kanazawa, 920-8641 Japan; 5 Division of Nephrology, Kanazawa Medical University School of Medicine, Uchinada, Ishikawa 920-0293 Japan

**Keywords:** Growth factor signalling, Mechanisms of disease

## Abstract

Peritoneal fibrosis (PF) is a severe complication of peritoneal dialysis, but there are few effective therapies for it. Recent studies have revealed a new biological function of trehalose as an autophagy inducer. Thus far, there are few reports regarding the therapeutic effects of trehalose on fibrotic diseases. Therefore, we examined whether trehalose has anti-fibrotic effects on PF. PF was induced by intraperitoneal injection of chlorhexidine gluconate (CG). CG challenges induced the increase of peritoneal thickness, ColIα_1_ mRNA expression and hydroxyproline content, all of which were significantly attenuated by trehalose. In addition, CG challenges induced a marked peritoneal accumulation of α-SMA^+^ myofibroblasts that was reduced by trehalose. The number of Wt1^+^ α-SMA^+^ cells in the peritoneum increased following CG challenges, suggesting that a part of α-SMA^+^ myofibroblasts were derived from peritoneal mesothelial cells (PMCs). The number of Wt1^+^ α-SMA^+^ cells was also suppressed by trehalose. Additionally, trehalose attenuated the increase of α-SMA and ColIα_1_ mRNA expression induced by TGF-β_1_ through Snail protein degradation, which was dependent on autophagy in PMCs. These results suggest that trehalose might be a novel therapeutic agent for PF through the induction of autophagy and the suppression of mesothelial-to-mesenchymal transition in PMCs.

## Introduction

Organ fibrosis is a common pathway that finally results in organ failure. Disease-related injuries are responsible for triggering fibrogenic responses. Fibrosis is a short-term adaptive response for wound healing, but prolonged injuries progress and lead to the overproduction of extracellular matrix (ECM). The dynamic deposition of ECM promotes progression to organ fibrosis and ultimately to organ failure^1^. Peritoneal fibrosis (PF) is a serious complication for patients undergoing peritoneal dialysis (PD)^2^, which is a life-sustaining therapy for patients with end-stage renal disease worldwide, which accounts for 11% of the overall dialysis population^3^. Dialysis solution is hyperosmotic and hyperglycemic, and can induce consecutive peritoneal injuries, thus inducing the progression of peritoneal fibrosis^4^. The development of PF causes encapsulating peritoneal sclerosis, which is a lethal complication of PD and an important problem that makes long-term PD difficult^2,5,6^. However, the precise molecular mechanisms for the development of PF need to be clarified to establish therapeutic strategies.

Accumulation of myofibroblasts, which have the capability of producing ECM and are characterized by α-smooth muscle actin (α-SMA) expression, is a critical process of tissue fibrosis^7^. Previous studies suggested that myofibroblasts can be derived from various cellular sources including resident fibroblasts, epithelial cells, mesothelial cells, endothelial cells, pericytes, and bone marrow derived cells^8–12^. In particular, epithelial/mesothelial cells have been known to differentiate into myofibroblasts through epithelial/mesothelial-to-mesenchymal transition (EMT/MMT). EMT/MMT is an established biological process by which stationary epithelial/mesothelial cells undergo phenotypic changes including the loss of cell–cell adhesion and apical-basal polarity and the acquisition of mesenchymal features such as α-SMA expression^13^. The Snail family zinc finger 1 (Snail1) is a transcription factor known to be a potent EMT/MMT inducer during embryonic development, fibrosis and tumor progression^14^. The expression of Snail1 has been reported to be upregulated predominantly by transforming growth factor-β (TGF-β) signaling during the progression of fibrosis, thereby repressing the expression of various important genes for maintaining the characteristics of epithelial/mesothelial cells, such as E-cadherin^15^. Therefore, targeting the TGF-β-Snail1 pathway might be a candidate to inhibit fibrosis development.

Autophagy is a well-known biological mechanism that degrades unnecessary and dysfunctional intracellular components^16^. A wide variety of stimuli, including nutrient deprivation, induce autophagy as an adaptive catabolic response. The expression of autophagy-related (Atg) proteins and the conversion of LC3-I to LC3-II promote the production of autophagosomes, which fuse with lysosomes and lead to protein degradation^17^. It has been previously reported that autophagy is related to diverse diseases including infection, cancer, aging, and neurodegenerative diseases in order to protect organisms^18^. A recent report has revealed that autophagy might be involved in the degradation of Snail and the inhibition of EMT^19^.

Trehalose (α-d-glucopyranosyl α-d-glucopyranoside) is a non-reducing disaccharide that consists of two 1,1-linked α-glucose monosaccharides. This sugar is present in a wide variety of organisms including plants, bacteria, yeast, fungi, insects, and invertebrates. In these organisms, trehalose can be a source of energy and protect intracellular proteins from a variety of stressful conditions including desiccation, dehydration, heat, cold, and oxidation^20^. Recent studies have revealed a new biological effect of trehalose as an autophagy inducer, and it can be considered to be a hopeful candidate as a therapeutic agent for neurodegenerative diseases through the enhancement of unnecessary protein degradation^21–23^. There are few reports concerning the therapeutic effects of trehalose by inducing autophagy on fibrotic diseases. In the current study, we found that trehalose attenuated PF through the suppression of myofibroblast accumulation derived from mesothelial cells in an in vivo model. Further, we found that trehalose promoted Snail1 degradation through induction of autophagy, thereby inhibiting MMT in response to TGF-β_1_, which could be a possible molecular mechanism. The current study suggests that trehalose could provide a beneficial therapeutic strategy to inhibit PF through the attenuation of MMT in PD patients.

## Results

### Trehalose protected mice from CG-induced PF

**Figure 1:**
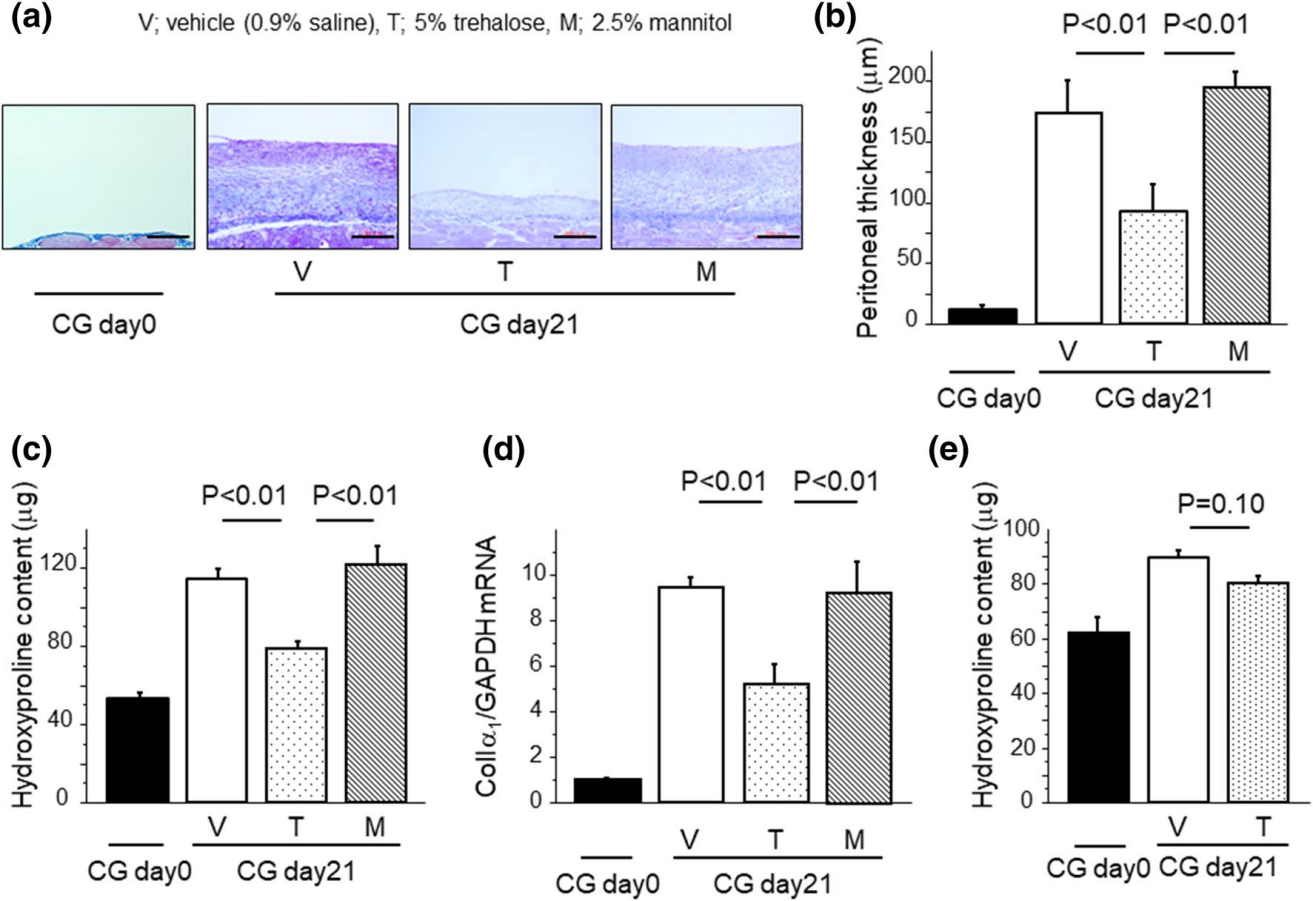
Trehalose protected mice from CG-induced PF. (**a**) Representative Mallory–Azan-stained peritoneal sections of mice following CG challenges (Day 21, magnification × 200). Bars, 100 μm. (**b**) Peritoneal thickness from mice following CG challenges (Day 21, n = 4 mice/group). (**c**) Hydroxyproline content in the peritoneum following CG challenges (Day 21, n = 9 mice/group). (**d**) Peritoneal expression of ColIα1 mRNA from mice following CG challenges (Day 21, n = 6 mice/group). (**e**) Hydroxyproline content in the peritoneum in the therapeutic regimen following CG challenges (Day 21, n = 5 mice/group). Data are expressed as mean ± SEM.

In order to clarify the effect of trehalose for PF, we administrated 5% trehalose solution (440 mOsm/L) into peritoneal cavity in chlorhexidine gluconate (CG)-induced PF model mice, and we also used 0.9% saline and 2.5% mannitol solution as control. The administration of 5% trehalose solution in mice showed its suppressive effects on CG-induced PF. Mallory–Azan staining demonstrated that collagen deposition induced by CG challenges was markedly reduced in the trehalose group compared with the vehicle (0.9% saline) and mannitol group (Fig. 1a). The degree of protection for PF was quantified by measuring peritoneal thickness, hydroxyproline content, and mRNA expression of α_1_ chain of type I pro-collagen (ColIα_1_). Peritoneal thickness followed by CG challenges was significantly reduced in the trehalose group compared with vehicle group (Fig. 1b). The increase in peritoneal hydroxyproline content observed in the vehicle group was suppressed in the trehalose group at day 21 (Fig. 1c). The increase in ColIα_1_ mRNA expression in the peritoneum was also significantly reduced after trehalose treatment (Fig. 1d). In contrast, any protective effects for PF were not observed in the mannitol group, which had the same osmolality (440 mOsm/L) as the trehalose group (Fig. 1a–d). Delayed administration of trehalose in the therapeutic regimen showed a decreasing trend in peritoneal hydroxyproline content, which was not statistically significant (Fig. 1e). These data indicate that trehalose has protective effects for PF through the reduction of collagen production and deposition by its biological mechanisms except for its osmolality.

### Trehalose contributed to the reduction of peritoneal myofibroblast accumulation during fibrogenesis

**Figure 2:**
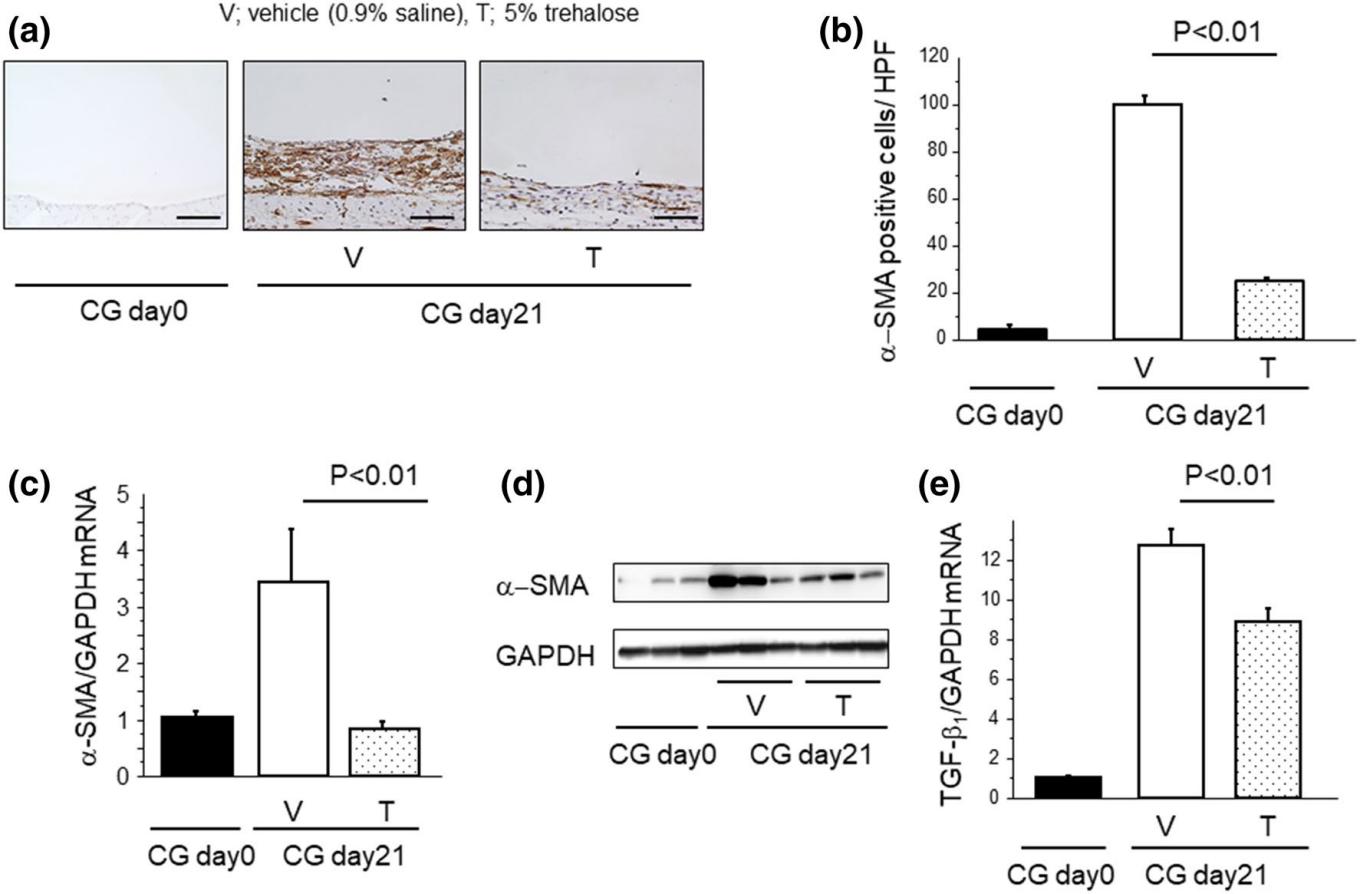
Trehalose contributed to the reduction of peritoneal myofibroblast accumulation during fibrogenesis. (**a**) The localization and expression of α-SMA protein in the peritoneum. Representative tissue sections of mice following CG challenges (Day 21) (magnification × 200). Bars, 100 μm. (**b**) Numbers of α-SMA positive cells in the peritoneum. Data are expressed as the mean number ± SEM per HPF (n = 3 mice/group). (**c**) Peritoneal expression of α-SMA mRNA at day 21 (n = 6 mice/group). (**d**) Peritoneal expression of α-SMA protein from mice following CG challenges (Day 21, n = 3 mice/group). (**e**) Peritoneal expression of TGF-β_1_ mRNA at day 21 (n = 6 mice/group). Data are expressed as mean ± SEM.

Myofibroblasts have been reported to accumulate prominently in areas of collagen deposition in a variety of fibrotic diseases; thus, myofibroblasts have been considered to be an important effector cell type for the production of ECM and the establishment of pathological fibrotic lesions^1^. We assessed the effect of trehalose on the peritoneal accumulation of myofibroblasts, which were identified as α-SMA expressing cells by immunostaining. As demonstrated by the representative peritoneal sections in Fig. 2a, CG challenges induced the robust α-SMA^+^ myofibroblast accumulation in the vehicle (0.9% saline) group. The accumulation of α-SMA^+^ myofibroblasts induced by CG challenges was significantly attenuated in the trehalose group (100.2 ± 3.7 cells/HPF vs. 24.7 ± 1.7 cells/HPF, respectively; Fig. 2b). The increase in peritoneal expression of α-SMA mRNA induced by CG challenges was similarly reduced by trehalose administration (Fig. 2c), as was observed in α-SMA protein expression (Fig. 2d). In addition, we have checked the expression of transforming growth factor (TGF)-β_1_. The increase in peritoneal expression of TGF-β_1_ mRNA induced by CG challenges was similarly reduced by trehalose administration (Fig. 2e). These data suggest that trehalose contributes to the reduction of peritoneal myofibroblast accumulation during fibrogenesis.

### Trehalose suppressed the accumulation of myofibroblasts derived from peritoneal mesothelial cells (PMCs)

**Figure 3:**
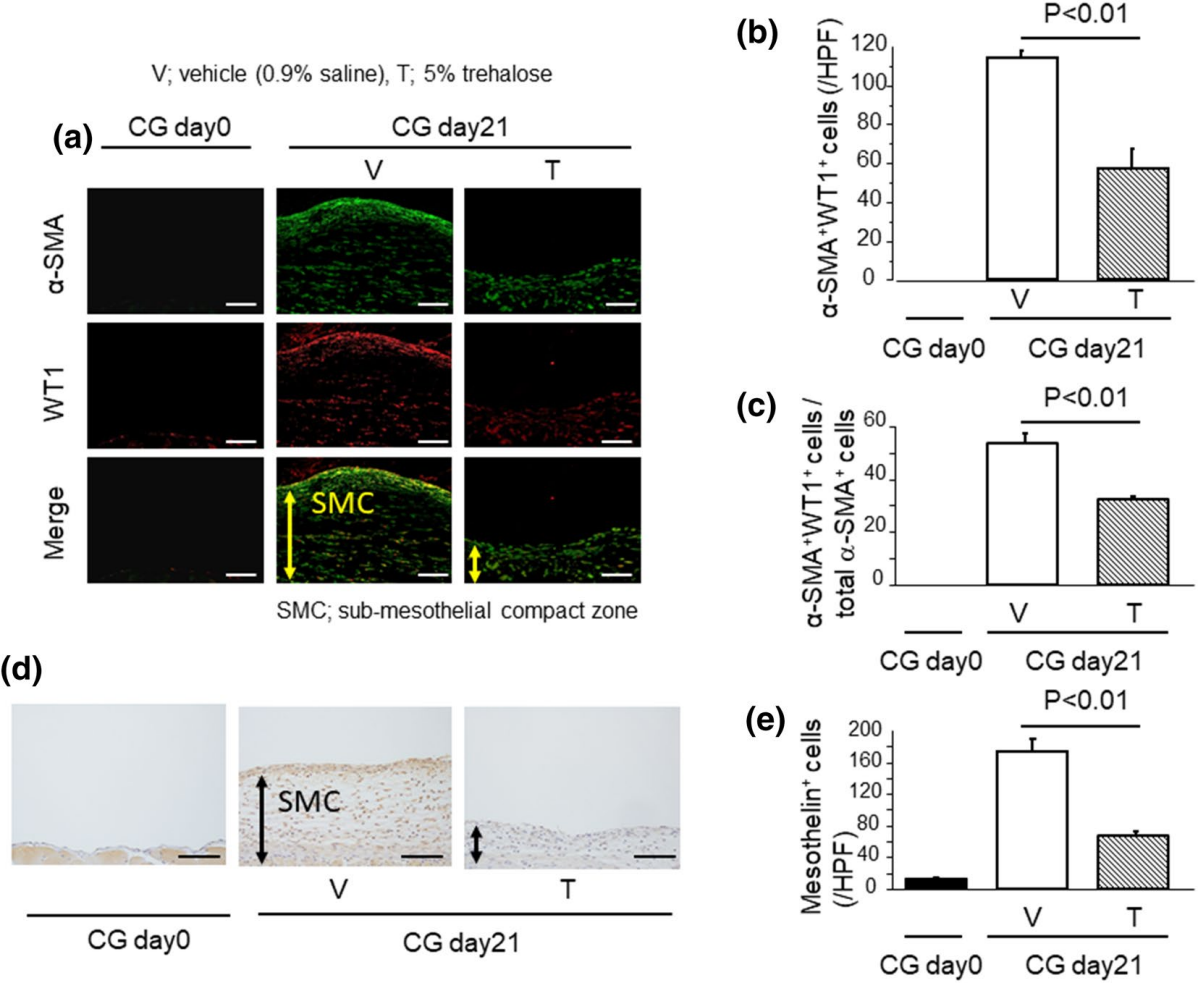
Trehalose suppressed the accumulation of myofibroblasts derived from PMCs. (**a**) Accumulation of myofibroblasts derived from PMCs (α-SMA^+^ Wt^+^, Wt1; Wilms tumor 1). Representative tissue sections of mice following CG challenges (Day 21) (magnification × 200). Bars, 100 μm. (**b**, **c**) Numbers of α-SMA^+^ WT1^+^ cells (**b**) and percentage of α-SMA^+^ WT1^+^/total α-SMA^+^ cells (**c**) in the peritoneum. (**d**) The localization of mesothelin protein in the peritoneum. Representative tissue sections of mice following CG challenges (Day 21) (magnification × 200). Bars, 100 μm. (**e**) Numbers of mesothelin-positive cells in the peritoneum. Data are expressed as the mean number ± SEM per HPF (n = 3 mice/group).

Various types of cells have been shown to be sources of myofibroblasts in fibrotic tissues^8–11^. Of these, PMCs have been known to be able to differentiate into myofibroblasts through MMT^10^. Therefore, to investigate whether PMCs were a source of myofibroblasts, we evaluated the number and distribution of Wilms tumor 1 (Wt1) and α-SMA dual positive cells during PF induced by CG challenges. Wt1 is known to be one of the markers for mesothelial cells. As demonstrated in the representative sections in Fig. 3a, Wt1^+^ cells were present in the mesothelial layer and these cells did not express α-SMA in control mice. In contrast, CG challenges induced a remarkable increase of Wt1 and α-SMA dual positive cells, especially abundant in the sub-mesothelial compact zone. In addition, the increase in the number of Wt1 and α-SMA dual positive cells was markedly reduced in the trehalose group (114.4 ± 2.9 cells/HPF vs. 57.2 ± 10.3 cells/HPF, respectively; Fig. 3b), as was the percentage of these dual positive cells among total myofibroblasts (percentage of Wt1 and α-SMA dual positive cells among total α-SMA^+^ cells): 53.5% ± 3.7% versus 32.3% ± 1.1%, respectively (Fig. 3c). In addition, we also performed immunostainings for mesothelin, which is another marker for mesothelial cells, using peritoneal samples. As shown in Fig. 3d, e, mesothelin-positive cells were abundant in surface layer as well as submesothelial compact zone in peritoneal samples. Therefore, we suggest that PMCs might be distributed throughout the fibrotic peritoneum after CG induction, and a part of myofibroblasts could be derived from PMCs in CG model. In addition, trehalose may contribute to the suppression of myofibroblast accumulation by inhibiting MMT of PMCs in a PF model. Taken together, we speculated that trehalose ameliorates MMT in PMCs, and the decrease of myofibroblast accumulation resulted in the suppression of PF.

### Trehalose reduced the expressions of α-SMA and ColIα_1_mRNA by the stimulation with TGF-β_1_ in PMCs

**Figure 4:**
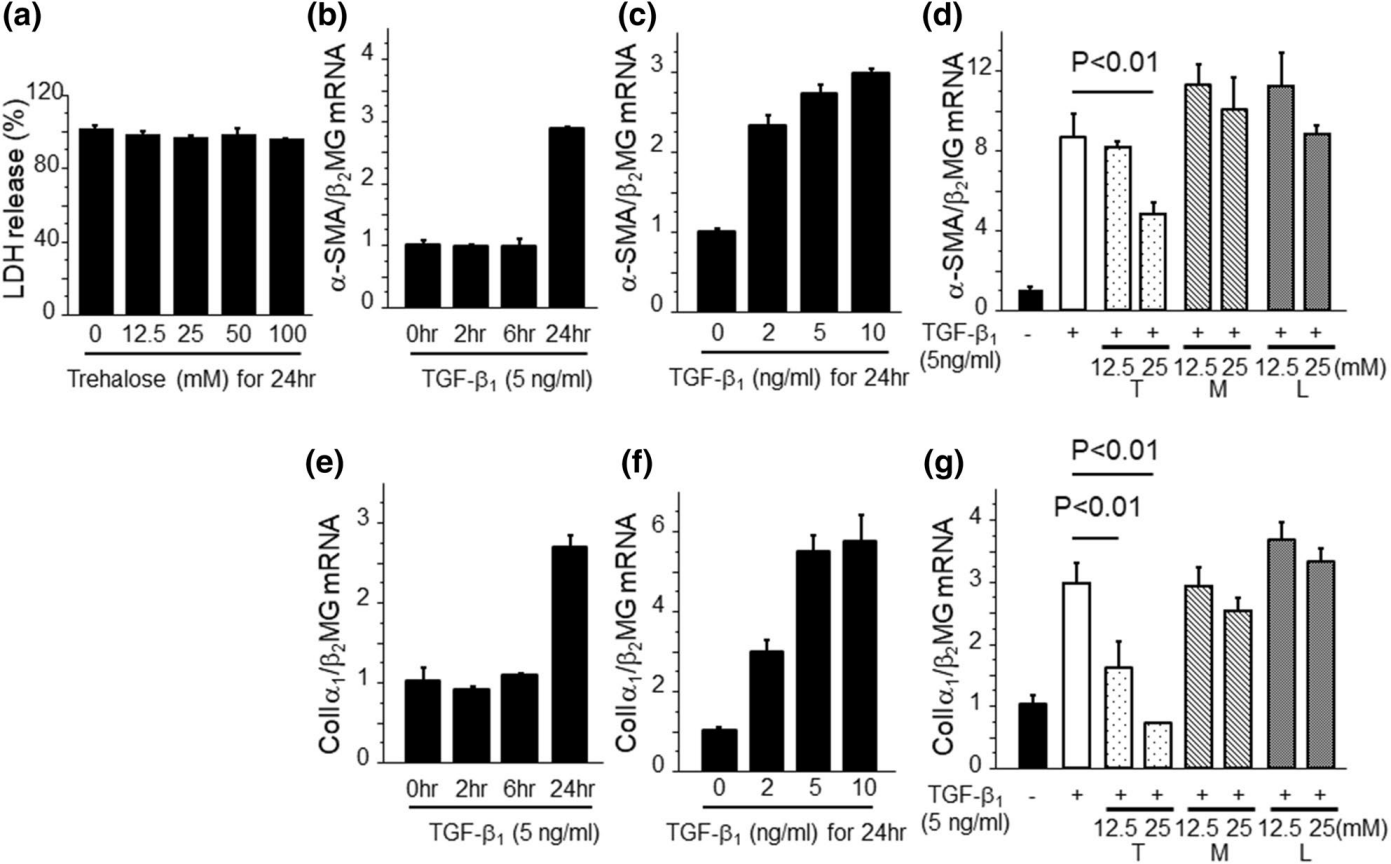
Trehalose reduced the expression of α-SMA and ColIα_1_mRNA expression by the stimulation with TGF-β_1_ in PMCs. (**a**) The LDH release of cells following 24 h incubation with trehalose. LDH release were assessed by absorbance at 490 nm and expressed as relative proportions to control samples without trehalose ± SEM (n = 3 cell preparation/group). (**b**, **c**) TGF-β_1_ induced α-SMA mRNA expression in a time- and dose-dependent manner (n = 3 cell preparation/group). (**d**) The effects of trehalose on TGF-β_1_-induced α-SMA mRNA expression (n = 3 cell preparation/group; T, trehalose; M, mannitol; L, l-glucose). PMCs were pre-treated with either trehalose, mannitol or l-glucose for 30 min prior to TGF-β_1_ stimulation. (**e**, **f**) TGF-β_1_ induced increase of ColIα1 mRNA expression in a time- and dose-dependent manner (n = 3 cell preparation/group). (**g**) The effects of trehalose on TGF-β_1_-induced ColIα_1_ mRNA expression (n = 3 cell preparation/group). PMCs were pre-treated with either trehalose, mannitol or l-glucose for 30 min prior to TGF-β_1_ stimulation. Data are expressed as mean ± SEM. Each experiment was performed two times independently.

Next, we performed in vitro studies to further validate the involvement of trehalose in MMT by PMCs, as was suggested by our in vivo studies. At first, we examined the cell toxicity of trehalose for PMCs by performing LDH assays. As shown in Fig. 4a, there was no significant increase of LDH release by trehalose for 24 h, suggesting that trehalose is not toxic to PMCs at a concentration of 100 mM. Next, we stimulated PMCs by TGF-β_1_ to evaluate the contribution of trehalose to MMT by evaluating α-SMA expression. TGF-β_1_ induced robust α-SMA mRNA expression in a time- and dose-dependent manner (Fig. 4b,c). This increase of α-SMA mRNA expression induced by TGF-β1 for 24 h was significantly reduced after pre-treatment with trehalose. In contrast, the reduction of α-SMA mRNA expression was not observed in PMCs pre-treated with mannitol or l-glucose, which had the same osmolality as trehalose (Fig. 4d). TGF-β_1_ also induced the increase in ColIα_1_ mRNA expression in a time- and dose-dependent manner (Fig. 4e, f). This increase in ColIα_1_ mRNA expression was reduced by trehalose, but not by mannitol or l-glucose (Fig. 4g). Taken together, trehalose attenuates MMT and ECM production induced by TGF-β_1_ in PMCs independent of osmolality.

### Snail1 protein expression was rapidly decreased by trehalose administration

**Figure 5:**
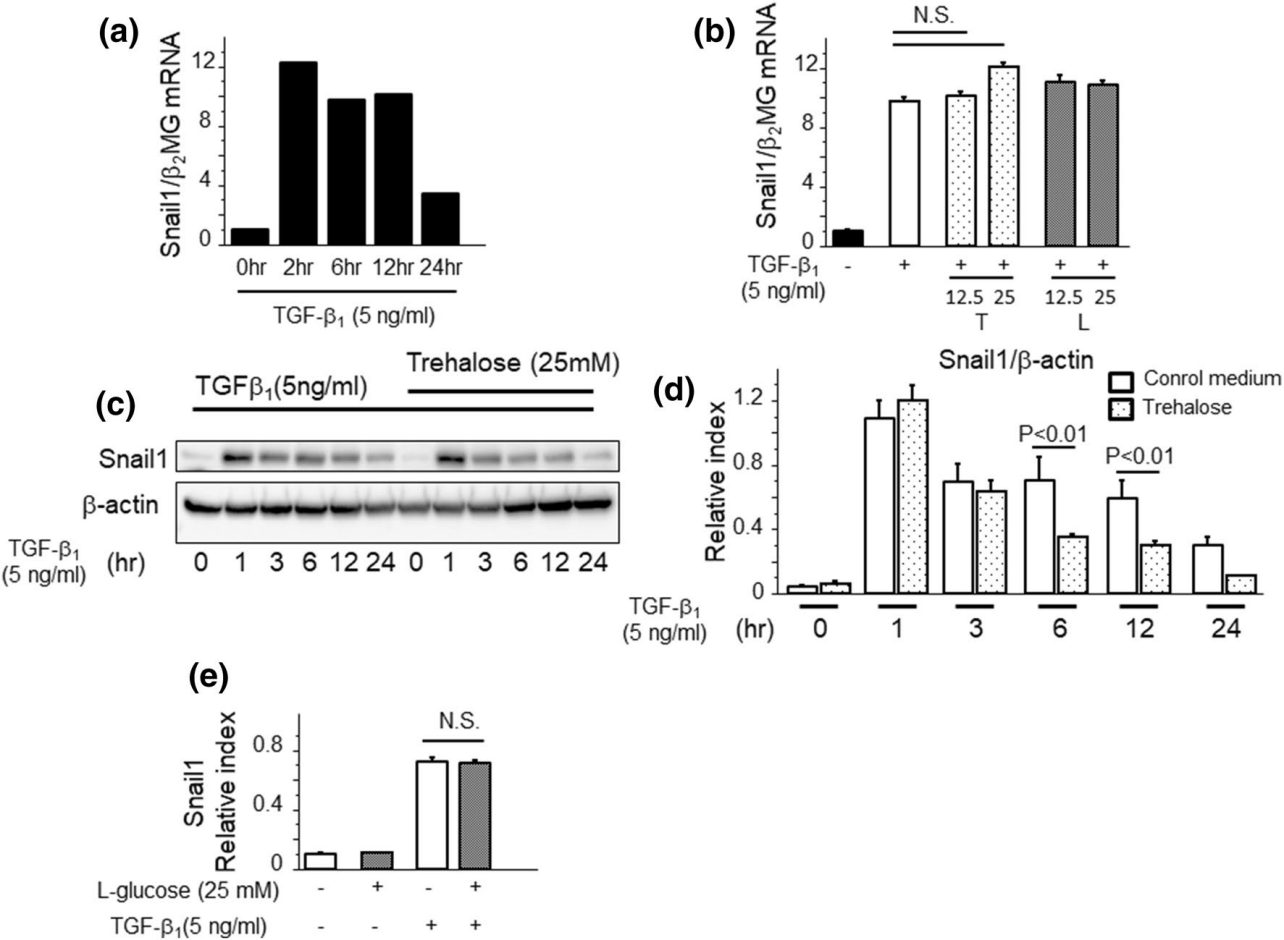
Snail1 protein expression was rapidly decreased by trehalose administration. (**a**) TGF-β_1_ promptly induced an increase of Snail1 mRNA expression. (**b**) The effects of trehalose on Snail1 mRNA expression induced by TGF-β_1_ for 2 h (n = 3 cell preparation/group, T; trehalose, L; l-glucose). PMCs were pre-treated with either trehalose or l-glucose for 30 min prior to TGF-β_1_ stimulation. (**c**, **d**) The effects of trehalose on Snail1 protein expression induced by TGF-β_1_ (n = 3 cell preparation/group). PMCs were pre-treated with trehalose for 30 min prior to TGF-β_1_ stimulation. (**e**) The effects of l-glucose on Snail1 protein expression induced by TGF-β_1_ (n = 3 cell preparation/group). PMCs were pre-treated with l-glucose for 30 min prior to TGF-β_1_ stimulation. Data are expressed as mean ± SEM. Each experiment was performed two times independently.

MMT is considered to be an important mechanism during PF^10,24^. TGF-β_1_ binds to TGF-β receptor and leads to the activation of the SMAD signaling pathway which induces Snail1 protein expression. Snail1 is a key regulator of MMT, which is characterized by the increase in the mesenchymal marker α-SMA expression and the decrease in the epithelial marker E-cadherin^15,24,25^. Therefore, we evaluated effects of trehalose on Snail1 expression in PMCs. TGF-β_1_ promptly induced robust Snail1 mRNA expression, (Fig. 5a); however, this expression was not altered by additional administration of trehalose (Fig. 5b). We then evaluated the time course of Snail1 protein expression induced by TGF-β_1_ using western blotting. TGF-β_1_ enhanced the expression of Snail1 1 h after stimulation and then this expression slowly decreased (Fig. 5c,d). In contrast, Snail1 expression decreased more rapidly by pre-treatment with trehalose (Fig. 5c,d). In Fig. 5e, we checked the effects of Snail1 protein degradation by l-glucose, however, l-glucose did not reveal reduction of Snail1 protein. Therefore, trehalose, not l-Glucose, may have impact on Snail1 degradation independent of osmolality. Taken together, these data indicate that trehalose ameliorate MMT because of its contribution to the degradation pathway of Snail1 at the post-transcription phase.

### Trehalose promoted the production of autophagosomes and suppressed MMT in PMCs

**Figure 6:**
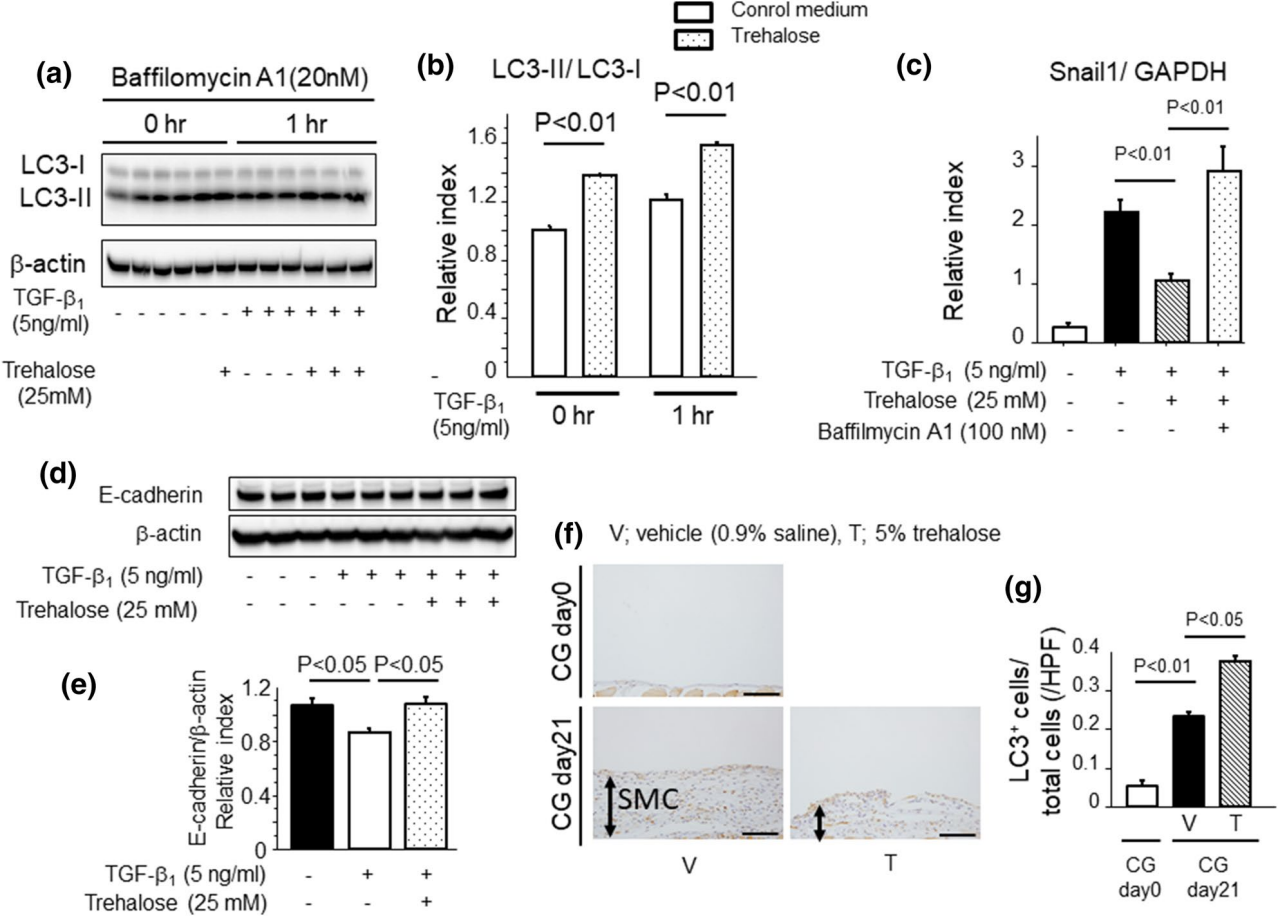
Trehalose promoted the production of autophagosomes and suppressed MMT in peritoneal mesothelial cells. (**a**, **b**) PMCs were pre-treated with baffilomycin A1 for 1 h followed by 30 min pre-treatment with trehalose. Then, TGF-β_1_ was added to PMCs for 1 h. Trehalose increased the ratio of LC3-II to LC3-I protein expression as compared with vehicle treatment. Data are expressed as mean density of LC3-II bands relative to LC3-I bands ± SEM expression (n = 3 cell preparation/group). (**c**) The inhibition of autophagy by bafilomycin A1 upregulated Snail1 protein expression even under the incubation with trehalose (n = 3 cell preparation/group). Data are expressed as mean ± SEM. (**d**, **e**) The effects of trehalose on E-cadherin protein expression in PMCs (n = 3 cell preparation/group). Data are expressed as mean density of E-cadherin bands relative to β-actin bands ± SEM. (**f**) The localization of GFP protein in the peritoneum in LC3-GFP mice. Representative tissue sections of mice following CG challenges (Day 21) (magnification × 200). Bars, 100 μm. (**g**) Numbers of GFP-positive cells in the peritoneum. Data are expressed as the mean number ± SEM per HPF (n = 3 mice/group).

As mentioned above, trehalose contributed to the reduction of Snail1 protein expression induced by TGF-β_1_ in PMCs. Therefore, we speculated that trehalose could influence intracellular protein degradation mechanisms. Two pathways that have been previously reported as Snail1 degradation pathways are related to autophagy and the proteasome^19,26^. Trehalose is well known to be an autophagy inducer in various cells, however, the effect of trehalose on autophagy in PMCs remains unclear. To confirm the effects of trehalose on autophagy in PMCs, we evaluated the accumulation of LC3-II which is positively related to the production of autophagosomes. In this investigation, we pre-incubated PMCs with bafilomycin A1, a V-ATPase (vacuolar-type H^+^ adenosine triphosphatase) inhibitor, to prevent LC3-II degradation in autophagosomes. Pre-treatment with trehalose increased the ratio of LC3-II to LC3-I protein expression in PMCs as compared to PMCs in control medium (Fig. 6a,b). These data suggest that trehalose induces the protein degradation pathway related to autophagy. Next, we evaluated the effect of autophagy on Snail1 protein expression. The inhibition of autophagy by bafilomycin A1 upregulated Snail1 protein expression even under the incubation with trehalose (Fig. 6c). This result suggests that Snail1 degradation could be regulated by autophagy induction in response to trehalose. In addition, the reduction of E-cadherin induced by TGF-β_1_ was reversed by pre-treatment with trehalose for 24 h in PMCs (Fig. 6d,e). To further confirm the involvement of autophagy in mice model of PF treated with trehalose, we investigated the expression of LC3 in peritoneum during fibrosis by using LC3-green fluorescent protein (GFP) transgenic mice treated with chloroquine as previously reported^27^. As shown in Fig. 6f,g, we observed that LC3 positive cells in peritoneum were increased in trehalose-treated CG group compared to 0.9% saline-treated CG group. Therefore, we suggest that trehalose might induce autophagy in CG-induced PF model.

### Trehalose had no effect on the protein degradation pathway related to the proteasome

**Figure 7:**
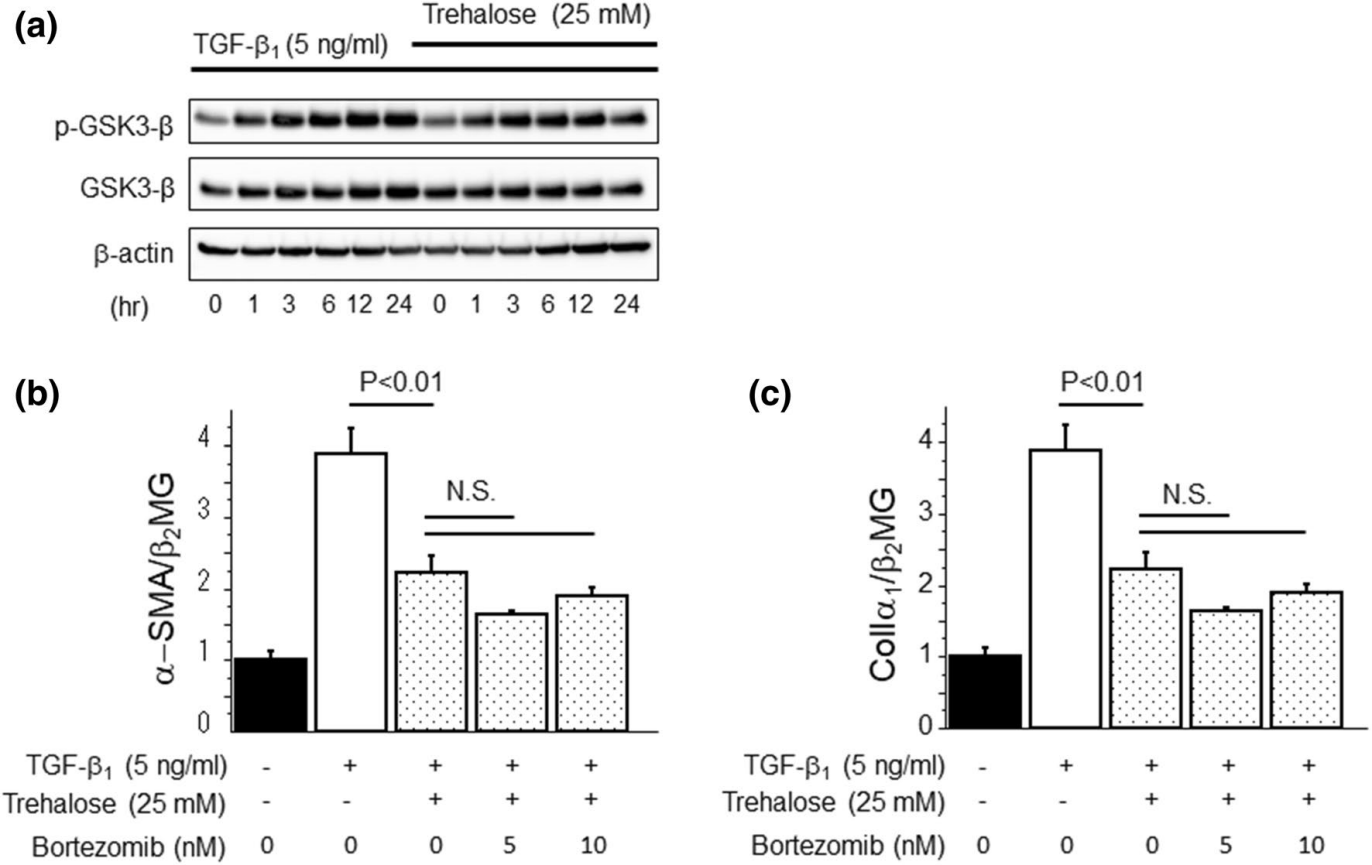
Trehalose had no effect on the protein degradation pathway related to the proteasome. (**a**) PMCs were pre-treated with trehalose for 30 min prior to TGF-β_1_ stimulation. Trehalose did not alter the ratio of phosphorylated GSK3-β to total GSK3-β protein expression induced by TGF-β_1_. Data are expressed as mean density of phosphorylated GSK3-β bands relative to total GSK3-β bands ± SEM expression (n = 3 cell preparation/group). (**b**, **c**) PMCs were pre-treated with bortezomib for 1 h followed by 30 min pre-treatment with trehalose. Then, TGF-β_1_ was added to PMCs for 24 h. Bortezomib did not influence the suppressive effects of trehalose on α-SMA and ColIα_1_ mRNA expression induced by TGF-β_1_in PMCs (n = 3 cell preparation/group). Data are expressed as copies of α-SMA and ColIα_1_ mRNA relative to copies of β_2_ microgloblin mRNA ± SEM. Each experiment was performed two times independently.

Next, we also evaluated the effects of trehalose on another protein degradation pathway related to the proteasome. In contrast to the pathway related to autophagy, trehalose had no effect on the phosphorylation of glycogen synthase kinase 3-β (GSK3-β) (Fig. 7a), which is a key molecule involved in the proteasome pathway. We performed another in vitro study with bortezomib, a proteasome inhibitor, to confirm the effects of trehalose on the protein degradation pathway related to the proteasome. However, bortezomib did not influence the suppressive effects of trehalose on α-SMA and ColIα_1_ mRNA expression induced by TGF-β_1_ in PMCs (Fig. 7b,c). Taken together, these data indicate that trehalose contributes to the suppression of MMT in PMCs independent of proteasome protein degradation pathway.

### Trehalose had no effect on SMAD or mitogen-activated protein kinase (MAPK) signaling pathways induced by TGF-β_1_

**Figure 8:**
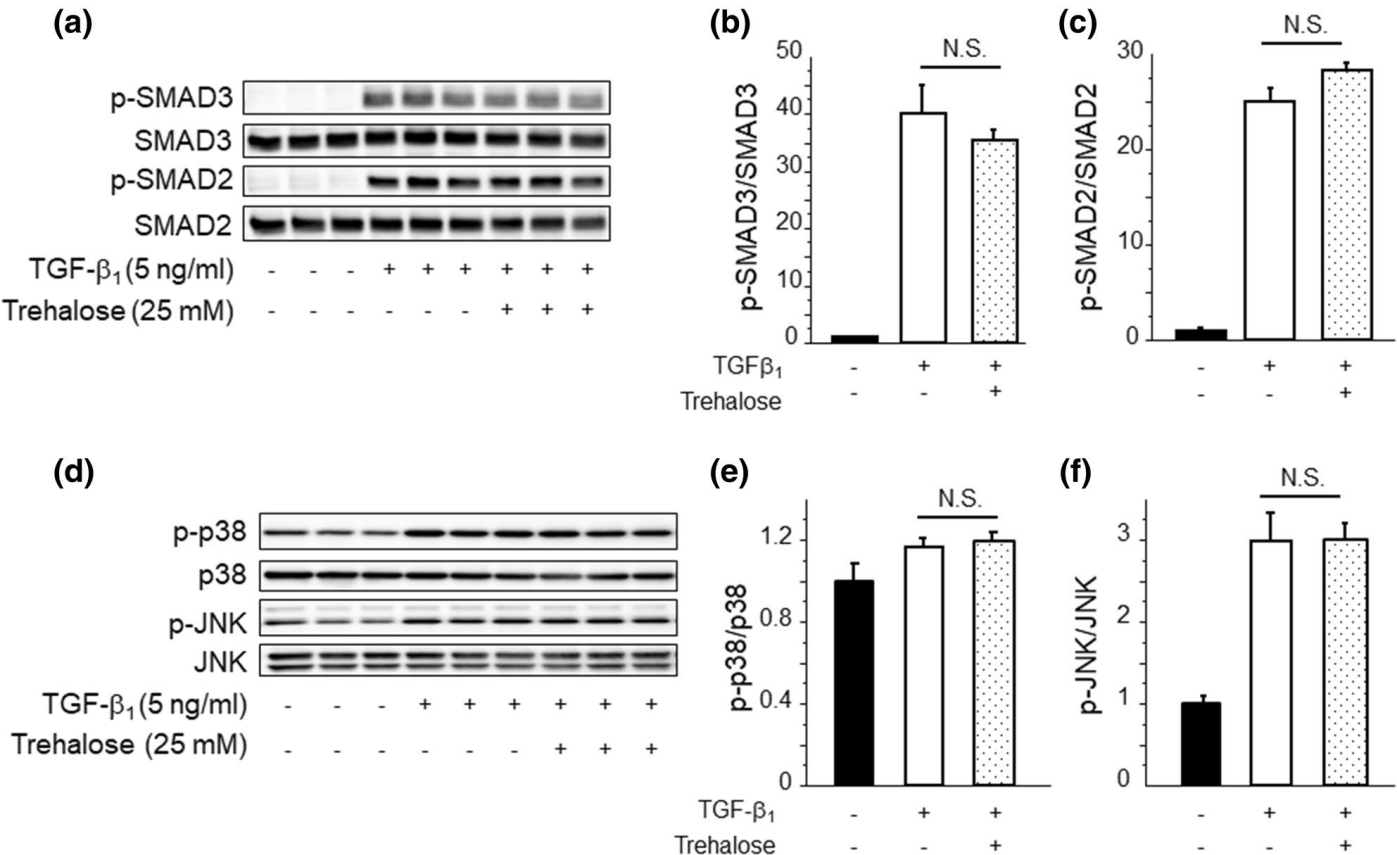
Trehalose had no effect on SMAD or MAPK signaling pathways induced by TGF-β_1_. (**a**–**c**) PMCs were pre-treated with trehalose for 30 min prior to TGF-β_1_ stimulation. The effects of trehalose on TGF-β_1_-induced SMAD phosphorylation were shown using cell lysate obtained from PMCs stimulated with TGF-β_1_ for 30 min (n = 3 cell preparation/group). Data are expressed as mean density of phosphorylated SMAD2/3 bands relative to total SMAD2/3 bands ± SEM. (**d**–**f**) PMCs were pre-treated with trehalose for 30 min followed by the stimulation with TGF-β_1_ for 30 min. The effects of trehalose on TGF-β_1_ induced MAPK phosphorylation (n = 3 cell preparation/group). Data are expressed as mean density of phosphorylated p38-MAPK bands relative to total p38-MAPK bands and phosphorylated JNK bands relative to total JNK bands ± SEM, respectively. Each experiment was performed two times independently.

In addition to Snail1 expression, it is well known that TGF-β_1_ directly regulates α-SMA and ColIα_1_ mRNA expression through various intracellular signaling pathways, including SMAD and MAPK signaling pathways^28–30^. p38 MAPK and JNK are well known important factors of non-canonical TGFβ_1_ signaling pathway which is crucial for organ fibrosis as with SMAD, therefore we needed to assess the relation between trehalose and activation of these pathways. At first, we evaluated the phosphorylation of Smad2/3; however, trehalose did not alter the phosphorylation of SMAD 2/3 induced by TGF-β_1_ (Fig. 8a–c) for 30 min. We also evaluated the phosphorylation of SMAD 2/3 induced by TGF-β_1_ at another time point (60 min), and there was no difference regarding trehalose effect on SMAD signaling (data not shown). Next, we evaluated the phosphorylation of MAPK, including p38 MAPK and JNK. Phosphorylation of p38 MAPK and JNK was increased by TGF-β_1_ for 30 min, but trehalose did not suppress the phosphorylation of these MAPK (Fig. 8d–f). These data suggest that trehalose might not be involved in SMAD or MAPK signaling pathways.

### Trehalose attenuated collagen producing fibroblast accumulation and proliferation in in vivo and in vitro studies

**Figure 9:**
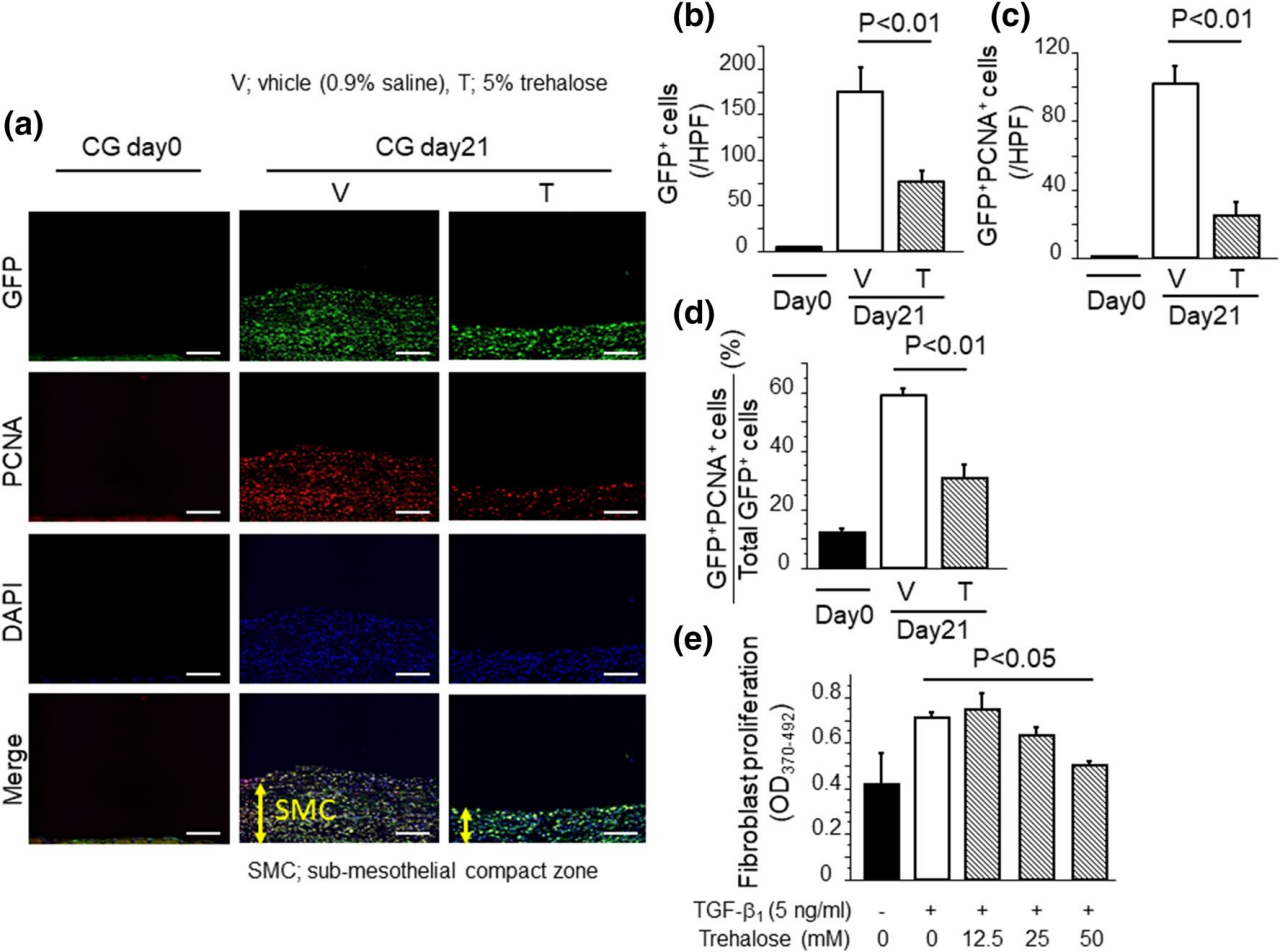
Trehalose attenuated collagen producing fibroblast accumulation and proliferation in in vivo and in vitro studies. (**a**) Peritoneal accumulation of proliferating fibroblasts after CG challenges. Representative tissue sections of mice following CG challenges (Day 21) (magnification × 200). Bars, 100 μm. (**b**) Numbers of sub-mesothelial GFP^+^ cells (fibroblasts), expressed as mean number per HPF (n = 3 mice/group). (**c**) Numbers of sub-mesothelial GFP^+^ PCNA^+^ cells (proliferating fibroblasts), expressed as mean number per HPF (n = 3 mice/group). (**d**) Percentage of sub-mesothelial fibroblasts that are proliferating (n = 3 mice/group). (**e**) NIH3T3 fibroblasts were pre-treated with control medium or trehalose at indicated concentrations for 30 min, and then incubated with TGF-β_1_. BrdU proliferation assays were performed after incubation with TGF-β_1_ for 48 h (n = 3 cells preparations/group), and expressed as mean OD value (OD_370–492_). Data are expressed as means ± SEM.

In addition to the importance of MMT in PF, a recent study has shown that the proliferation of fibroblasts in the sub-mesothelial zone also plays an important role in PF^31^. Therefore, we examined fibroblast proliferation induced by CG challenges in ColIα_2_-enhanced green fluorescent protein (GFP) transgenic mice. GFP positive cells means pro-collagen producing cells, which we considered that these cells might be a mixture of MMT mesothelial cells as well as fibroblasts. As demonstrated in the representative sections in Fig. 9a, CG challenges induced a remarkable peritoneal accumulation of GFP^+^ fibroblasts that was significantly reduced by trehalose. The increased number of GFP^+^ fibroblasts that were induced by CG-challenges in the vehicle (0.9% saline) group, was markedly reduced by trehalose administration (Fig. 9b). In order to specifically identify proliferating fibroblasts, we double-stained peritoneal sections with anti-PCNA antibody and anti-GFP antibody. As demonstrated in the representative sections in Fig. 9a, fibroblast proliferation induced by CG challenges was also ameliorated by trehalose. The number of PCNA and GFP dual positive proliferating fibroblasts in the peritoneal tissue after CG challenges was significantly increased in the vehicle group as compared to the trehalose group (101.5 ± 10.6 cells/HPF vs. 24.3 ± 7.9 cells/HPF, respectively; Fig. 9c), as was the percentage of proliferating fibroblasts among total fibroblasts (percentage of PCNA and GFP dual positive cells among total GFP^+^ cells): 58.7% ± 2.4% versus 30.4% ± 4.9%, respectively (Fig. 9d). We next performed an in vitro study to confirm the effects of trehalose on the proliferation of fibroblasts (NIH3T3). TGF-β_1_ induced fibroblast proliferation; however, trehalose suppressed the fibroblast proliferation in a dose-dependent manner (Fig. 9e). These data suggest that trehalose also ameliorates fibroblast proliferation and contributes to the inhibition of myofibroblast accumulation during PF.

## Discussion

**Figure 10:**
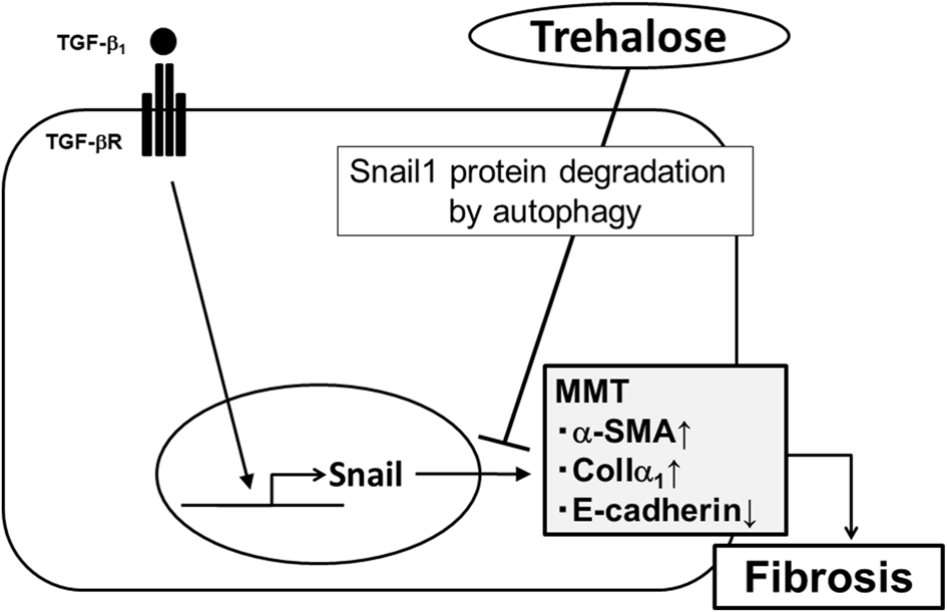
Proposed schema for the effects of trehalose on PF. Trehalose contributes to the promotion of Snail1 degradation by induction of autophagy and ameliorates MMT in PMCs. Therefore, trehalose might be an effective therapeutic reagent for PF.

In this study, we found that trehalose ameliorated PF through the induction of autophagy and the suppression of MMT. Trehalose protected mice from PF induced by repetitive CG challenges. The number of α-SMA expressing myofibroblasts in the peritoneum increased with the development of PF. Trehalose reduced the number of α-SMA^+^ myofibroblasts in fibrotic peritoneum induced by CG challenges, and also reduced the number of Wt1^+^ α-SMA^+^ myofibroblasts which were considered to be derived from PMCs. In vitro studies using mouse PMCs demonstrated that trehalose reduced the increase of α-SMA and ColIα_1_ mRNA expression induced by TGF-β_1_ through the suppression of MMT in PMCs. Trehalose enhanced Snail1 degradation by promoting the protein degradation pathway related to autophagy. Taken together, these data indicate that trehalose contributes to the amelioration of PF by regulating myofibroblast accumulation dependent on MMT (Fig. 10).

Previous reports have revealed that PMCs were one of the important sources of myofibroblasts during PF through their transition to a mesenchymal phenotype^10,32,33^. Animal models have clarified that high glucose levels and TGF-β_1_ induced PF through MMT in PMCs^10,34^. In addition, it has been revealed that human mesothelial cells purified from PD fluid underwent MMT through the Snail1 expression induced by TGF-β_1_^33^_._ Taken together, the regulation of MMT inducers, including glucose and TGF-β_1_, might be a prospective therapeutic option for peritoneal fibrosis. In daily clinical settings, we have been using glucose as an osmolyte of PD fluid, which contains high concentrations of glucose for ultrafiltration of water. However, a high concentration of glucose in PD fluid has been known to induce PF through the production of glucose degradation products (GDPs), advanced glycation end products and various cytokines/chemokines^34–36^_._ Trehalose is a non-reducing disaccharide and could function as an osmolyte of PD fluid. Our results showed that trehalose contributed to the attenuation of peritoneal fibrosis by inhibiting MMT in response to TGF-β_1_. Therefore, trehalose could be a safer osmolyte of PD fluid and a therapeutic compound for PF.

This study showed that trehalose induced autophagy, thereby suppressing MMT through Snail1 degradation. Especially, the effect of autophagy by trehalose showed earlier than Snail1 degradation in this study, suggesting that autophagy induction by trehalose was preceded to Snail1 degradation. Recent reports revealed that trehalose was an autophagy inducer^36^, and had therapeutic effects on neurodegenerative diseases through unnecessary protein degradation, such as α-synuclein and prion protein^21–23^. Several pathways have been reported in various cells for the mechanism through which trehalose regulates autophagy. For instance, trehalose has been reported to induce autophagy in hepatocytes through inhibition of intracellular glucose transportation and activation of 5′-adenosine monophosphate (AMP)-activated protein kinase (AMPK) signaling, which phosphorylated unc-52-like kinase-1 (ULK1) at Ser^317^^37,38^. In addition, it has also been reported that activation of AMPK signaling induced autophagy through another pathway which was related to attenuation of mammalian target of rapamycin 1 complex (mTORC1) signaling and decrease of the phosphorylation of the inhibitory site of ULK1 (Ser^757^)^37^. Our results suggest that trehalose is capable of inducing autophagy and promoting Snail1 degradation in PMCs, therefore, clarifying the downstream pathways of trehalose that regulate autophagy might be a useful strategy to develop new therapeutic targets to combat PF.

The proliferation of resident fibroblasts in the sub-mesothelial zone has been considered to be important for myofibroblast accumulation resulting in the progression of PF^31^. We showed that trehalose attenuated the proliferation and accumulation of fibroblasts in fibrotic peritoneum induced by repetitive CG challenges in an in vivo study and also suppressed the proliferation of NIH3T3 induced by TGF-β_1_ in an in vitro study. Snail1 expression is considered to be involved in cell survival through activating mitogen-activated protein kinase kinase 7 (MAP2K7)/extracellular signal-regulated kinase (Erk) and phosphoinositide 3-kinase (PI3K)/serine/threonine specific protein kinase (Akt) pathways^39,40^. A recent report has also suggested that Snail1 expression was associated with cell proliferation in fibroblasts^41^. Therefore, proliferation and accumulation of fibroblasts might be related to the function of Snail1, which was suppressed by trehalose, suggesting the potential of trehalose as an anti-fibrotic agent targeting fibroblast/myofibroblast biologies. On the other hand, GFP positive cells in ColIα_2_-GFP mice means pro-collagen producing cells, which we considered that these cells might be a mixture of resident fibroblasts as well as collagen-producing PMCs after MMT. Future investigation will be required to identify which type of cell has more contribution to the production of collagen.

Trehalose has been utilized for protection against desiccation through abundant intracellular accumulation which leads to crypto-biosis in some insects^42^. In addition, trehalose transporter 1 (TRET1) was recently isolated from *polypedilum vanderplanki*, and it was reported that TRET1 was involved in a trehalose-specific transportation dependent on the concentration gradient of trehalose^42^. Even in humans, glucose transporter 8 (GLUT8) has amino acid sequence homology with TRET1, and also has the capability of trehalose intracellular transportation in a recent report^38^. We presume that trehalose was also transported into the cytoplasm at least in part through GLUT8 and induced autophagy in PMCs. Future investigations will be required to clarify the mechanisms of trehalose uptake in PMCs during the course of PF.

In terms of the importance of MMT regulated autophagy in PF, there have been controversial results. Wu et al.^43^ reported that the involvement of MMT in human peritoneal mesothelial cells (HPMCs). In that report, they only performed in vitro studies using HPMCs, and they stimulated HPMCs with high glucose. However, we clarified that trehalose suppressed peritoneal fibrosis through the induction of autophagy in mice model of peritoneal fibrosis using LC3-GFP mice. In addition, Grassi et al.^19^ also showed that the promotion of autophagy attenuated EMT through Snail1 degradation in hepatocytes. Taken together, especially based on our results, autophagy induction by trehalose administration might be involved in the protection from CG-induced PF.

Besides the involvement of Snail1 in MMT, SMAD and MAPK such as p38 MAPK and JNK are also known important factors to induce MMT, therefore we assessed the relation between trehalose and activation of these pathways. However, based on our results, trehalose might have no impact for SMAD and MAPK signaling pathway. Taken together, we speculate that trehalose could affect Snail1, not SMAD or MAPK, thereby contributing to the inhibition of MMT through Snail1 degradation.

We have limitation of this study, because we could not evaluate the net peritoneal ultrafiltration of trehalose in this mice model. We consider that trehalose might be a candidate as an osmolyte for peritoneal dialysis. Furthermore, we could not evaluate the effect of Snail1 overexpression on Trehalose-induced inhibition of MMT by technical difficulties. To address these issues, future investigations will be required.

In summary, we have shown that trehalose suppressed PF by degrading Snail1 and suppressing MMT through induction of autophagy. Our results suggest that trehalose could be a prospective therapeutic compound for PF and an osmolyte of PD fluid, which is important for safe continuation of long term PD. In addition, the effect of trehalose on suppression of MMT might be applied to other fibrotic diseases that are associated with EMT/MMT.

## Materials and methods

### Reagents and cells

Trehalose (α-d-glucopyranosyl α-d-glucopyranoside) was purchased from Hayashibara (Okayama, Japan) and d-Mannitol and l-glucose were purchased from Wako Pure Chemical Industries (Osaka, Japan). All of them were diluted in Dulbecco’s modified Eagle’s medium (DMEM; Thermo Fisher Scientific, Waltham, MA) which included 0.5% fatty acid-free bovine serum albumin (BSA; Thermo Fisher Scientific). l-glutamine, Sodium pyruvate, NEAA mixture, and penicillin/streptomycin were obtained from Thermo Fisher Scientific. Baffilomycin A1 and Bortezomib were purchased from Sigma Aldrich Japan (Tokyo, Japan). Recombinant TGF-β_1_ was from R&D Systems (Minneapolis, MN). NIH3T3 fibroblasts were purchased from American Type Culture Collection (Manassas, VA). In each in vitro experiment, we use DMEM with 10% fetal bovine serum (FBS) for cell culture, and 12 h starvation was performed using DMEM without FBS followed by administration of reagent or osmotic agent.

### Mice

We purchased male C57BL/6 mice (8 weeks, 20–25 g) obtained from Charles River Japan (Atsugi, Japan). Experiments to identify fibroblasts used ColIα_2_-enhanced GFP transgenic mice generated on the C57BL/6 background (a kind gift from Dr. Yutaka Inagaki, Tokai University, Isehara, Kanagawa, Japan). In order to evaluate the autophagic flux in vivo, we used LC3-GFP mouse strain (RBRC00806) which was provided by RIKEN BRC through the National Bio-Resource of the MEXT, Japan^44^. All experiments used sex- and weight-matched mice at 8–10 weeks of age that were maintained in specific pathogen-free environments. All procedures used in the animal experiments complied with the standards set out in the guidelines for the care and use of laboratory animals of Kanazawa University, and were approved by the Institute for Experimental Animals, Kanazawa University Advanced Science Research Center (registration number: AP-143227).

### Peritoneal fibrosis model

PF was induced by intraperitoneal injection of 0.1% chlorhexidine gluconate (CG) (Wako Pure Chemical Industries) dissolved in 15% ethanol/phosphate buffered saline (PBS) as previously described^45^. CG was injected in mice every other day over a period of 21 days.

### Trehalose administration in vivo

Trehalose was dissolved in 0.9% saline. Doses of 1.5 ml 5% trehalose solution were administered by intraperitoneal injection to mice every other day when mice did not receive CG injection. Control mice received equal volumes of 0.9% saline alone (vehicle) or 2.5% mannitol solution on the same schedule. 0.9% saline, trehalose solution or mannitol solution was administered beginning on the day following the onset of CG challenge in a preventive regimen, or beginning 5 days after the onset of CG challenges in a therapeutic regimen. In the experiment assessing the autophagic flux in vivo, chloroquine (Sigma-Aldrich; 50 μg/g body wt) was injected intraperitoneally and mice were euthanized 6 h after the injection^27^.

### Histology and peritoneal thickness measurement

A sample of peritoneal tissue from each mouse was fixed in 10% buffered formalin (pH 7.2) and embedded in paraffin. We stained 5-μm sections with Mallory–Azan stain. Peritoneal thickness was measured from the junction of the parietal peritoneum with the musculature of the abdominal wall to the serosal surface of the parietal peritoneum, as described previously^45^. Measurements were done on photomicrographs (× 200) of Mallory–Azan stained sections at five randomly selected sites per high-power field (HPF) per section.

### Hydroxyproline assay

Two pieces of peritoneal samples were taken from each mouse [6-mm punch biopsy apparatus (Kai Corporation, and Kai Industries Co., Tokyo, Japan)] to assess peritoneal collagen, measured by its hydroxyproline content as determined by the standard protocol of our laboratory^44^. Assay results were expressed as micrograms (μg) of hydroxyproline per piece.

### RNA analyses

Total cellular RNA was isolated from cultured cells using a High Pure RNA Isolation Kit (Roche Diagnostics K.K., Tokyo, Japan). Total cellular RNA was isolated from peritoneal tissues by immersing the surface of the peritoneum in Trizol reagent (Thermo Fisher Scientific) for 20 min and then extracting RNA with the Pure link RNA kit (Thermo Fisher Scientific) according to the manufacturer’s instructions. Quantitative real-time PCR analyses of RNA using ViiA™ 7 (Thermo Fisher Scientific) was performed for the detection of ColIα_1_, α-SMA, TGF-β_1_ and Snail1. Glyceraldehyde-3-phosphate dehydrogenase (GAPDH) and β_2_ microglobulin (β2MG) were used as PCR controls. Data are expressed as mean ± SEM. Primer sequences were listed in Table 1.

**Table 1:** Primer sequences.

Mouse αSMA-F: 5′-CTGACAGAGGCACCACTGAA-3′
Mouse αSMA-R: 5′-CATCTCCAGAGTCCAGCACA-3′
Mouse β2MG-F: 5′-CCGAACATACTGAACTGCTACG-3′
Mouse β2MG-R: 5′-CCCGTTCTTCAGCATTTGGA-3′
Mouse GAPDH-F: 5′-CAACTACATGGTCTACATGTTCCAGT-3′
Mouse GAPDH-R: 5′-TGACCCGTTTGGCTCCA-3′
Mouse COL1α1-F: 5′-CTCCTCTTAGGGGCCACT-3′
Mouse COL1α1-R: 5′-CCACGTCTCACCATTGGGG-3′
Mouse SNAIL1-F: 5′-CACACGCTGCCTTGTGTCT-3′
Mouse SNAIL1-R: 5′-GGTCAGCAAAAGCACGGTT-3′
Mouse TGFβ1-F: 5′AGCAACAATTCCTGGCGTTACC-3′’
Mouse TGFβ1-R: 5′AGTGAGCGCTGAATCGAAAGC-3′

### Immunohistochemical analyses

For the current analysis, formalin-fixed, paraffin-embedded sections were prepared as described above. α-SMA expressing cells were identified using anti-mouse α-SMA polyclonal antibodies (DAKO, Santa Clara, CA). Mesothelin expressing cells were identified using anti-mouse mesothelin monoclonal antibodies (Abcam, Cambridge, MA). To assess levels of autophagic flux in peritoneal tissue in vivo study, we used LC3-GFP mice and anti-mouse GFP monoclonal antibodies (Cell Signaling, Danvers, MA). α-SMA, mesothein and GFP positive cells were visualized by incubating antibody-stained sections with DAB (DAKO), respectively. To identify the source of α-SMA^+^ cells, dual immunostainings with anti-Wt1 antibodies (Abcam) and anti-α-SMA monoclonal antibody (Abcum) were performed using peritoneal sections from C57BL/6 mice. To identify proliferating fibroblasts, peritoneal sections from Col1α_2_-enhanced GFP mice were co-stained with anti-mouse GFP monoclonal antibody (Cell Signaling) and anti-mouse PCNA monoclonal antibody (Abcam), using an M.O.M. kit (Vector laboratories, Burlingame, CA). Antibody-stained cells were visualized using Fluorescein avidin (Vector laboratories) and Texas-red avidin (Vector laboratories). Positive cells were then counted in all fields of the sub-mesothelial zone and expressed as the mean number ± standard error of the mean (SEM) per HPF.

### Western blot analyses

Whole cellular lysates from primary cells were extracted with RIPA buffer (Thermo Scientific) according to the manufacturer’s protocol. In brief, whole-tissue lysates were extracted from peritoneal samples by immersing the surface of peritoneal tissues in RIPA buffer (Thermo Scientific) for 20 min. Cellular and tissue lysates, and were then separated by SDS-PAGE and transferred to polyvinylidene difluoride (PVDF) membranes (Thermo Scientific). After incubation in PVDF blocking reagent (Toyobo, Osaka, Japan), membranes were incubated overnight at 4 °C with mouse anti-human α-SMA monoclonal antibody (Abcam), rabbit anti-mouse Smad2 monoclonal antibody (Cell Signaling), rabbit anti-mouse phospho-Smad2 monoclonal antibody (Cell Signaling), rabbit anti-mouse Smad3 monoclonal antibody (Cell Signaling), rabbit anti-mouse phospho-Smad3 monoclonal antibody (Cell Signaling), rabbit anti-mouse Snail monoclonal antibody (Cell Signaling), rabbit anti-mouse LC3A/B monoclonal antibody (Cell Signaling), rabbit anti-mouse JNK monoclonal antibody (Cell Signaling), rabbit anti-mouse phospho-JNK monoclonal antibody (Cell Signaling), rabbit anti-mouse p38-MAPK monoclonal antibody (Cell Signaling), rabbit anti-mouse phospho-p38MAPK monoclonal antibody (Cell Signaling), rabbit anti-mouse GSK3-β monoclonal antibody (Cell Signaling), rabbit anti-mouse phospho-GSK3-βmonoclonal antibody (Cell Signaling), rabbit anti-mouse β-actin monoclonal antibody (Cell Signaling) or anti-muse GAPDH monoclonal antibody (Cell signaling). Following additional incubations of the membranes with appropriate biotinylated secondary antibodies, protein bands were detected with an enhanced chemiluminescent substrate (Thermo Scientific). Quantification was performed with Image J software (NIH, Bethesda, MD, USA).

### Isolation of PMCs

Primary PMCs were isolated from mice by enzymatic digestion of the inner surface of the peritoneum, as described previously^46^, with minor modifications. Briefly, parietal-peritoneal flaps were removed and stretched on a sterile culture dish. RPMI 1,640 (Gibco BRL), containing 25 μg/ml Liberase (Roche, Basel, Switzerland), was placed on the peritoneal inner surface for 45 min at 37 °C. After incubation, the surface of the digested peritoneum was gently scraped to complete the release of partially attached mesothelial cells. The cell pellet was collected by centrifugation and re-suspended in growth medium composed DMEM with 20% FBS. To identify the outgrowing cells as mesothelial cells, immunocytochemistry with anti-vimentin antibody (VIM13.2, Sigma Aldrich) and anti-cytokeratin antibody (PCK-26, Sigma Aldrich) was performed. More than 98% of these cells were positive for vimentin and cytokeratin, consistent with their being mesothelial cells.

### LDH assay

Cytotoxicity of trehalose for PMCs was evaluated colorimetrically by the activity of the lactate dehydrogenase (LDH) released in the media. This assay was performed using the cytotoxicity LDH Assay Kit (Dojindo, Kumamoto, Japan) in accordance with the manufacturer’s protocol. PMCs were incubated with DMEM with 0–100 mM trehalose for 24 hr.

### Fibroblast proliferation assay

NIH3T3 fibroblasts were pre-treated with trehalose (0, 12.5, 25, 50 mM) for 1 h and then stimulated with 5 ng/ml TGF-β_1_ for 48 h. Fibroblast proliferation was determined by the BrdU assay (Roch, Mannheim, Germany) according to the manufacturer’s protocol.

### Statistical analyses

Data are expressed as means ± SEM. Unpaired *t* tests were used for comparison between two groups, and analysis of variance (ANOVA) with post hoc Fisher’s test was used for comparison between more than two groups. *P* values < 0.05 were considered statistically significant.
